# Immunizing Action of Iodide of Potassium in Regard to Aphthous Fever (Foot and Mouth Disease)1Abstracted by Dr. Otto Noack, Reading, Pa., from Revue Vétérinaire.

**Published:** 1896-05

**Authors:** 


IMMUNIZING ACTION OF IODIDE OF POTASSIUM IN REGARD TO APHTHOUS FEVER (Foot and Mouth Disease).[1] M. Piok (*Centralblatt für Bakteriologie*, 1895, No. 11) wishing to procure milk containing iodine for the treatment of syphilitic children, selected two cows to be given iodide of potassium in daily doses of twelve grammes. The result on these animals was an augmentation of thirst, and of the salivary and lacteal secretions. The milk contained a considerable quantity of iodine, which was also found in the urine of the children nourished by the milk. It happened that aphthous fever was brought among the milch-cows (numbering seventy). To shorten the duration of the epizoötic, virulent matter was taken from the diseased and put

[1] Abstracted by Dr. Otto Noack, Reading, Pa., from Revue Vétérinaire.

in the mouth of all animals still healthy. The aphthous fever appeared in all cows with the exception of the two which were taking the iodide of potassium. As they never had been attacked by the disease, and were kept under the same conditions of nourishment and hygiene as the others, their immunity is attributed to the iodide of potassium.

